# Wnt Signaling Inhibits High-Density Cell Sheet Culture Induced Mesenchymal Stromal Cell Aging by Targeting Cell Cycle Inhibitor p27

**DOI:** 10.3389/fbioe.2020.00946

**Published:** 2020-08-05

**Authors:** Ying Xu, Ye Tian, Dongyi Tong, Hao Zhang, Zhengliang Luo, Xifu Shang, Yufeng Dong

**Affiliations:** ^1^Department of Anesthesiology, Shengjing Hospital of China Medical University, Shenyang, China; ^2^Department of Orthopedics, Shengjing Hospital of China Medical University, Shenyang, China; ^3^Center for Tissue Engineering and Regenerative Medicine, Department of Orthopedic Surgery, Louisiana State University Health Sciences Center, Shreveport, LA, United States; ^4^Division of Life Sciences and Medicine, Department of Orthopedic Surgery, The First Affiliated Hospital, University of Science and Technology of China, Hefei, China

**Keywords:** aging, cell sheet culture, senescence, apoptosis, stromal cell

## Abstract

Mesenchymal stromal cell senescence and apoptosis have been identified as critical molecular hallmarks in aging. In this study, we used stromal cell sheet culture as an *in vitro* model to study the progressive changes of cellular senescence, apoptosis and underlying mechanism in Wnt3a treated cells. Our results showed fresh bone marrow mesenchymal stromal cells (BMSCs) become senescent and undergo apoptosis with increased inflammatory profile and Reactive Oxygen Species (ROS) in high-density cell sheet cultures. The gene expression level of senescence related proteins and key regulators of apoptosis in cell sheet cultures was significantly increased in older BMSCs at Days 4 and 7 cultures compared with younger cells at Day 1 cultures. More importantly, Wnt signaling activation significantly reduced senescence in cell sheet cultures by direct regulation of cell cycle inhibitor p27. This study not only characterized the cellular and molecular features of aging stromal cells in short-term cell sheet cultures, but also identified the downstream target responsible for Wnt inhibition of cell senescence.

## Introduction

Aging is a complex phenomenon and major risk factor for a variety of degenerative diseases, while its fundamental mechanism remain unclear. It has been suggested that stromal cell dysfunction plays an important role in this process (Signer and Morrison, 2013). Stromal cells are capable of self-renewal and differentiation to mature somatic cells *in vitro* and *in vivo*. In normal health conditions, tissue-specific stromal cells maintain tissue homeostasis and supply different progenitor cells for tissue regeneration after injury. During aging, stromal cells progressively lose their plasticity and stemness leading a progressive depletion of the stromal cell pool (Van and Liang, 2003). The reduced tissue-specific stromal cell number and activity has important consequences for the maintenance of tissue function that leads to multiple age-related diseases such as decreased immunity, delayed wound healing, muscle weakness, and osteoporosis (Bengal et al., 2017). Therefore, it becomes crucial to understand the dysfunction of stromal cell responses to aging requiring comprehensive mechanistic studies of the molecular events that influence stromal cell survival.

The process of aging has been characterized by showing different hallmarks at cellular, molecular, and organ levels. One major contributor to aging is the development of cellular senescence (Flatt, 2012; López-Otín et al., 2013). It is well-known that senescence cell permanently exits the cell cycle by showing senescence-associated secretory phenotype (SASP). SASP associated inflammatory factors promote immune cells infiltration resulting unbalanced local chronic inflammation that impairs tissue homeostasis (Childs et al., 2014; Soto-Gamez and Demaria, 2017; Zoico et al., 2019). *In vitro*, cellular senescence manifests the replicative exhaustion of cultured normal diploid cells (Hayflick and Moorhead, 1961). Phenotypically, senescent cells increase in size and protein content, with enlarged nuclei and lysosomes that possess elevated senescence-associated β-galactosidase activity (SA-β-Gal) (Young et al., 2009; Höhn et al., 2017). This marker is one of the most widely used for the identification of senescence in cell cultures and tissues (Kurz et al., 2000). Importantly, experimental clearance of cellular senescent cells in mice has been found to delay aging-related pathologies in some tissues further confirming its critical role in cell aging (Baker et al., 2011).

Regulation of stromal cell phenotype and cell aging involves many signaling factors, including the Wnt pathway. The Wnt signaling pathway is a complex cascade important in many fundamental cellular processes including cell fate determination during embryogenesis, cell proliferation, cell cycle arrest and differentiation, as well as apoptosis and tissue homeostasis (Clevers et al., 2014). While previous research has shown that activating the Wnt signaling pathway delays the rapid aging process and prolongs the viability of stromal cells in culture (Willert et al., 2003), the underlying mechanism of Wnt signaling on the cell sheet culture-induced cell senescence has not been assessed. The goal of this study was to develop an easy-setup cell culture system to study the effect of the Wnt signaling on stromal cell aging in regular culture conditions in 37°C incubator with 5% carbon dioxide (CO_2_) and 20% oxygen (O_2_).

## Materials and Methods

### BMSC Culture

Fresh human bone marrow derived stromal cells (BMSCs) were purchased from Lonza and designated for our purposes as Passage 0 (P0) cells. Cells were seeded at 5000 cells/cm^2^ in T75 flasks in 25 ml of BMSC growth medium (MSCGM^TM^, Lonza) until ∼80% confluence for expansion in regular culture conditions. A full media exchange was performed on Day 3 only if additional time in culture was needed to meet the desired confluence. Expanded BMSCs were harvested as Passage 1 (P1) and a portion of these cells were cultured in plates at high density (800 cells/mm^2^) for 24 h to form cell sheet as we described previously (Shang et al., 2017). The cell sheet cultures were harvested at Days 1, 4, and 7 for multiple assays described below. For treatment experiments, the BMSC sheets were cultured in media containing human Wnt3a (50 ng/ml; H17001, Sigma-Aldrich) for 3 days by changing media every 24 h prior to cell harvesting for protein, RNA extraction and proliferation assay.

### BrdU Labeling

Cell proliferation assays were performed using a BrdU ELISA Kit (Roche) as we described previously (Tian et al., 2017). Briefly, P1 BMSCs in cell sheet at Days 1, 4, and 7 were first exposed to BrdU labeling reagent for 6 h, and then incubated with FixDenat buffer for 30 min. After reaction with anti-BrdU-POD working solution, absorbance values were measured by a multi-mode microplate reader (BioTek Instruments) at 450 nm.

### ELISA Assays

Human IFNγ, IL-1β, and TNF-α sandwich ELISA kit from Abcam was used to determine the amount of IFNγ, IL-1β, and TNF-α in the BMSCs at cell sheet cultures as per the manufacturer’s instructions.

### β-Galactosidase Staining

Senescent cells were stained by using a Senescence Detection Kit (Cell Signaling Technology, Boston, MA) as described before (Zoico et al., 2019). Briefly, cell sheets cultured in a 6-well plate were first fixed with 1ml fixative solution for 10min at room temperature and 1ml of β-Galactosidase Staining Solution was added to the plate and incubated for 5h at 37°C protected from light. Cells were then counterstained with ready-to-use nuclear fast for 40s. Cells were photographed using an EVOS phase-contrast microscope (Advanced Microscopy Group, Bothell, WA). The percentage of β-gal positive cells was determined by counting positive and total cells in 5 randomly selected fields of view.

### Analysis of Apoptotic Cells

The cell apoptosis was detected by the Annexin V FITC apoptosis detection kit (BD Biosciences, San Jose, CA, United States) as we described previously (Xu et al., 2018). Briefly, on Days 1, 4, and 7 after cell sheet culture, the cells were washed with PBS and trypsinized. After centrifugation and washing, the cells were fixed in cold 70% ethanol and stained with propidium iodide (PI) and FITC Annexin V. The cell apoptosis percentage was analyzed by a flow cytometry machine. All the data were analyzed using FlowJo software (Tree Star).

### Analysis of ROS

The Intracellular Reactive Oxygen Species (ROS) level was measured using an Image-iT^TM^ LIVE Green Reactive Oxygen Species (ROS) Detection Kit (Thermo Fisher Scientific, United States) with a published protocol (Zoico et al., 2019). Cell sheets cultured in chamber slides were washed with warm HBSS and incubated with 25 μM carboxy-H2DCFDA working solution for 30min at dark. Hoechst 33342 was then added at a final concentration of 1.0 μM to the carboxy-H2DCFDA staining solution. Cells were then washed in HBSS and mounted with the coverslip for observation. Cells were observed in an EVOS FL Auto Cell Imaging System with EVOS Onstage Incubator (Thermo Fisher Scientific, United States) photomicroscope. Optical density (OD) assay was performed on 10 randomly chosen areas (20 μm^2^ draw each) positive for ROS by observing images at 200 × magnification using ImageJ software (National Institutes of Health, Bethesda, Maryland).

### Real Time RT-PCR

Total RNA was extracted from BMSC sheets using RNeasy^®^ mini kit by Qiagen as we previously detailed (Tian et al., 2017). DNA was synthesized from 1 μg total RNA using the SuperScript III reverse transcriptase kit (Invitrogen) in a final volume of 20 μl. RT-PCR experiments were performed in a Bio-Rad C1000 thermal cycler (Bio-Rad, Hercules, CA). Pairs of sequence-specific primers were designed to amplify a small DNA fragment (120–150 bp) for Cyclin D1: forward, 5′-ATGGAACATCAGCTGCTGT-3′, and reverse, 5′-TCAGATGT CCACATCCCGC-3′; p21: forward, 5′-GCCTGGACTGTTTT CTCTCG-3′, and reverse, 5′-ATTCAGCATTGTGGGAGGAG-3′; p16: forward, 5′-CACGGGTCGGGTGAGAGT-3′, and reverse, 5′-CCCAACGCACCGAATAGTTAC-3′; p53: forward, 5′-CTGTCATCTTCTGTCCCTTC-3′ and reverse, 5′-TGGAA TCAACCCACAGCTGCA3′; Bax: forward, 5′-TGGCAGCTGAC ATGTTTTCTG AC-3′ and reverse, 5′-TCACCCAACCACCC TGGTCTT-3′; Caspase-3: forward, 5′-GCAGCAAACCTCAG GGAAAC-3′ and reverse, 5′-TGTCGGCATACTGTTTCAGCA-3′; Bcl-2: forward, 5′-ATGTGTGTGGAGAGCGTCAACC-3′ and reverse, 5′-TGAGCAGAGTCTTCAGAGACAGCC-3′; p27 forward, 5′-GGCCTCAGAAGACGTCAAAC-3′; and reverse, 5′-CATGTATATCTTCCTTGCTTCATCA-3′. β-catenin, forward 5′-GTGCTATCTGTCTGCTCTAGTA-3′, reverse 5′-CTTCCTG TTTAGTTGCAGCATC-3′. Expression levels were normalized to internal control β-Actin: forward 5′-ATCTGGCACCACAC CTTCTA-3′ and reverse, 5′-CGTCATACTCCTGCTTGCTG-3′. Relative expression levels were calculated using the formula 2-(ΔΔCt). Each analysis was conducted at least in triplicate.

### Nuclear Extracts Preparation and Electrophoretic Mobility Shift Assay (EMSA)

Wnt3a-treated BMSCs were washed with PBS and nuclear proteins extracted from cells as previously described (Dong et al., 2006). Individual oligonucleotides were 5′−end [−32P]deoxyadenine triphosphate−labeled with T4 polynucleotide kinase. Labeled oligonucleotides (100 ng) were annealed to equimolar amounts of their complementary strands. EMSA was performed as described previously (Dong et al., 2006). Briefly, 20,000 cpm of labeled oligoduplex probes were added to 10 μg of nuclear extracts in 20 μl of DNA binding buffer (10% glycerol, 1 mM EDTA, 1 mM dithiothreitol, and 60 mM KCl). To prevent non−specific binding of nuclear proteins, poly (dI−dC) was added to a concentration of 100 ng/μl, and the binding reaction was incubated at room temperature for 30 min prior to electrophoresis. For competition experiments, wild−type or mutated oligonucleotides were added to nuclear extracts for 20 min at room temperature then incubated with labeled probe for 30 min before separation. Protein−DNA complexes were separated from unbound DNA using a 4.5% (w/v) polyacrylamide, 0.5 × Tris−borate EDTA non−denaturing gel at a constant voltage of 200 V for 2.5 h. Gels were visualized by autoradiography.

### Chromatin Immunoprecipitation Assay (ChIP)

Chromatin immunoprecipitation assay was performed as described earlier (Rezazadeh et al., 2019). The immune complexes were captured using protein A −Sepharose beads. After a series of washing steps, the beads were extracted in 500 μl of elution buffer (0.1 m NaHCO_3_, 1% SDS) and analyzed by PCR for β−catenin recruitment on the p27^Kip1^ promoter. Primers used for PCR amplification (5′ to 3′) were as follows: 1. p27 F, CTTTTGGCTCCGAGGGCA, and p27 R, CCGGGTCTGCAGCACCG spanning the region −137 to −7 relative to translation start site on the p27^Kip1^ promoter. 2. p27 F, CAAGGTTTGGAGAGCG, and p27 R, CGGAGAGGAGAGGGGACCAG spanning the region −345 to −238 relative to translation start site on the p27^Kip1^ promoter.

### Statistical Methods

Data are presented as means ± standard error (*SE*) for each experiment. Differences between groups were analyzed by ANOVA. A *p* < 0.05 was used to determine statistical significance.

## Results

### Rapid Cellular Aging Is Induced in Stromal Cell Sheet Cultures

**FIGURE 1:**
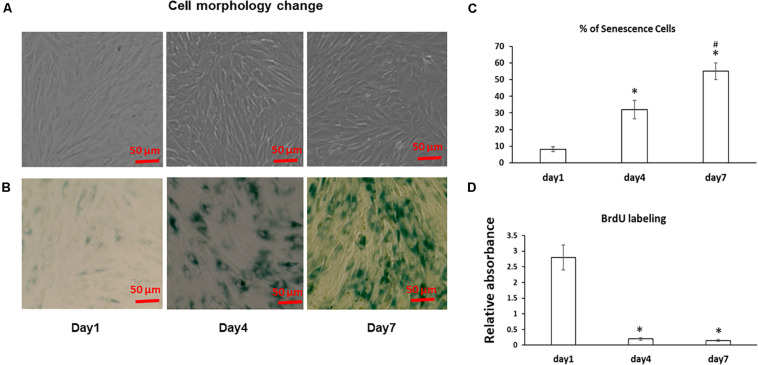
Cell morphology change and aging in BMSC sheet cultures. **(A)** BMSCs were cultured in cell sheets at Days 1, 4, 7 and visualized by microscope. Scale bar, 100 μm. **(B)** SA-β-gal staining was performed to determine the extent of senescence in 1, 4, and 7 Day cell sheet cultures. Scale bar represents 100 μm. **(C)** SA-β-gal positive cells were quantitated by ImageJ. **(D)** Cell proliferation was determined by BrdU labeling in cell sheet cultures at Days 1, 4, and 7. All experiments were performed in triplicate. *P < 0.05 vs. Day 1 cells and ^#^*P* < 0.05 vs. Day 4 cells.

The morphology of the stromal cells in cell sheet culture was first examined using a microscope. As shown in Figure 1A, fresh BMSCs at Day 1 had an elongated spindle shape, while larger, flatter cells were observed at Day 4 and 7 suggesting progressive changes in cell size from Day 1 to 7 in cell sheets cultures. Because bone marrow stromal cell aging is associated with accelerated senescence (Hayflick and Moorhead, 1961), we next measured the senescence associated β-galactosidase (SA-β-gal) activity, the hallmark of cellular senescence, in cell sheet culture for up to 7 Days. As shown in Figure 1B, very few MSCs are positive for SA-β-gal in the Day 1 cultures; however, the number of SA-β-Gal positive cells in the population increased from low levels (8.9 ± 2%) at the Day 1 cultures to 32 ± 5.5% at Day 4 cultures and reached the highest level of 55 ± 4.9% at Day 7 cultures (Figure 1C), suggesting cell sheet cultures induce a rapid aging in fresh BMSCs. Cell proliferation assay by BrdU labeling further showed most of the BMSCs entered cell cycle arrest from Day 1 to Day 4 indicating a quick stop of cell proliferation in cell sheet cultures (Figure 1D). Similar results were also observed in BMSCs isolated from additional two donors.

**FIGURE 2:**
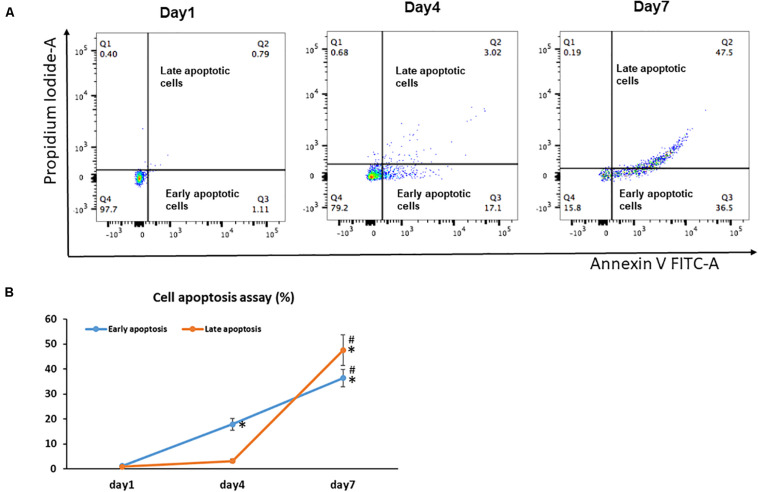
Cell apoptosis in BMSC sheet cultures. Cells from Days 1, 4, and 7 cultures were stained with Annexin V and PI, and the populations corresponding to viable and non-apoptotic (Annexin V– PI–), early (Annexin V+PI–), and late (Annexin V+PI+) apoptotic cells. **(A)** Representative flow cytometry graphs of cell apoptotic analysis. **(B)** The percentage of early apoptotic cells and the percentage of late apoptotic cells in Days 1, 4, and 7 cultures. Results are mean ± SD. All experiments were performed in triplicate. *P < 0.05 vs. Day 1 cells and *^#^P* < 0.05 vs. Day 4 cells.

Unlike senescent cells, which are permanently withdrawn from the cell cycle and viable to secrete pro-inflammatory factors, apoptotic cells enter programmed cell death and are permanently eliminated (Baar et al., 2017). Because increased apoptosis is also one of the pathognomonic characteristics of aging in tissues, we next examined the effect of cell sheet culture on cell apoptosis. The flow cytometry results showed very few apoptotic cells in the Day 1 cell cultures (Figure 2A). However, more early apoptotic cells appeared in the Day 4 cell sheet cultures, and more late apoptotic cells were observed in the Day 7 cell sheet cultures when the highest number of senescence cells were actually detected (Figure 2B).

**FIGURE 3:**
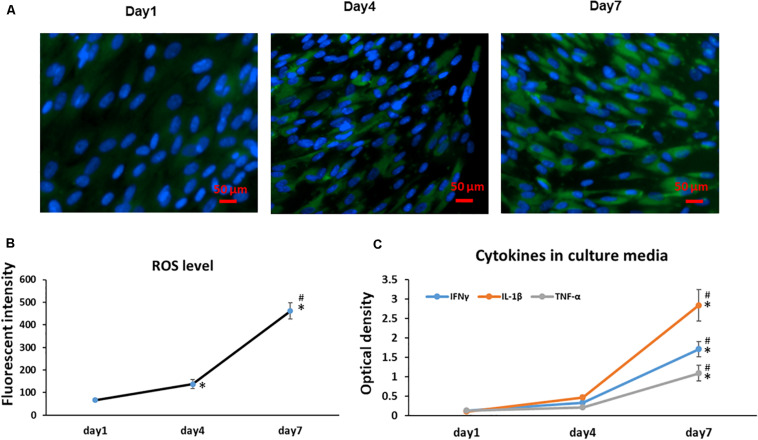
Cell sheet culture increases ROS production in BMSCs. **(A)** Representative immunofluorescence imagines of ROS formation (green fluorescent), and Hoechst 33342 (blue) was used to detect the nucleus in BMSCs at the indicated Days (scale bars: 50 μm). **(B)** Quantification of ROS level was achieved by calculation of the OD on randomly chosen areas (green positive), observing images at 200× magnification by Image J. *P < 0.05 vs. Day 1 cells and ^#^*P* < 0.05 vs. Day 4 cells. **(C)** Cytokines IFNγ, IL-1β, and TNF-α increased rapidly in BMSC sheet culture medium from Day 1 to 7. *P < 0.05 vs. Day 1 cells. All experiments were performed in triplicate.

Previous results have shown cellular senescence occurs when a high oxidative level is reached and prolonged (Colavitti and Finkel, 2005); thus, ROS have been described as an important mediator for cellular senescence progression. Similarly, with a greater number of aging MSCs observed at Day 4 and 7, we found the Day 7 cells showed a significantly increased accumulation of intracellular ROS when compared to cells at Day 1 and 4 cultures (Figure 3A); Interestingly, the intracellular ROS increased more in our culture system at later cultures from Day 4 to 7 (*p* < 0.001) compared to early cultures from Days 1 to 4 (Figure 3B). Since cellular senescence is marked by the SASP, we further measured the presence of three key cytokines in the cell culture medium at different time points. With increased time in stromal cell sheet cultures, we observed a rapid increase of secreted IFNγ, IL-1β, and TNF-α in the culture medium of BMSC sheet cultures from Day 1 to 7. Particularly, the IL-1β had the greatest increase compared to IFNγ and TNF-α (Figure 3C).

**FIGURE 4:**
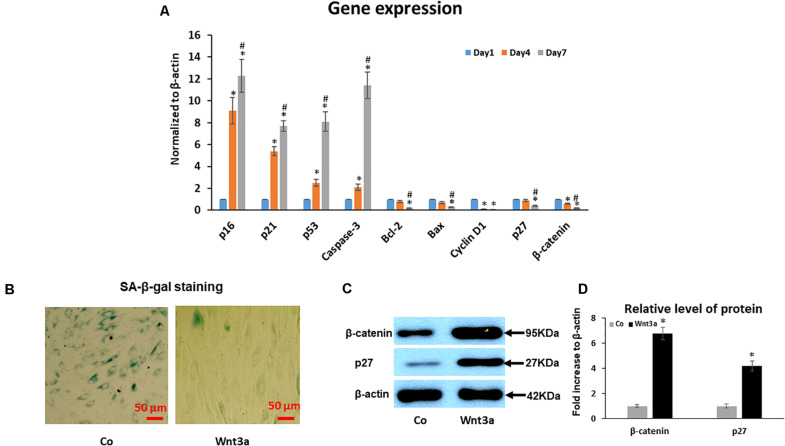
Effect of cell sheet culture on senescence and apoptosis related gene expression. **(A)** Expression of cell aging related genes was tested by Real-time PCR using total RNA isolated from BMSC sheets, including p16, p21, p53, p27 and Caspase-3. Anti-aging gene expression of Bcl-2, Bax, Cyclin D1, and β-catenin. *P < 0.05 vs. Day 1 cells and ^#^*P* < 0.05 vs. Day 4 cells. All experiments were performed in triplicate. **(B)** SA-β-gal staining showed that 3-day Wnt3a treatment reduced cell senescence in cell sheet cultures. **(C)** Western blot showed an increase of p27 and β-catenin in BMSCs after treatment with Wnt3a for 3 days. **(D)** Quantification of western blot bands shown in **(C)**.

To further identify the molecular pathways involved in the increased senescence in this cell sheet culture model of aging, we performed PCR for several key genes related to the activation of senescence and apoptosis pathways. PCR results showed that gene expression of cell cycle inhibitor p53, p21, and p16 increased from younger Day 1 cells to aged Day 4 and 7 cells (Figure 4A). Although our PCR data clearly showed an increased trend for mRNA expression of some senescence-associated genes, apoptosis associated gene caspase-3 was also significantly increased, specifically from Day 4 to 7. In contrast, anti-apoptotic gene Bcl-2, Bax and Cyclin D1 showed a deceased trend in aged MSCs from Day 4 to 7 cells when compared to young cells at Day 1 sheet cultures (Figure 4A). Surprisingly, cell cycle inhibitor p27 showed a decreased expression from Day 1 to 7 in sheet culture, which is not consistent with other cell cycle inhibitors showed above suggesting a unique role of p27 in regulation of cell sheet culture induced senescence in culture. Interestingly, expression of Wnt signaling effector β-catenin was decreased in culture by showing a similar expression pattern to p27. This result strongly suggests that Wnt signaling may interact with p27 to regulated cell death in BMSCs.

### Wnt3a Treatment Inhibits Cell Sheet Culture-Induced Senescence

To study the effect of Wnt signaling on cell senescence, we performed SA-β-gal staining and western blot assays in cell sheet culture treated with Wnt signaling ligand Wnt3a for 3 days. SA-β-gal staining clearly showed a reduced cell senescence in Wnt3a treated BMSCs (Figure 4B). More importantly, the western blot results showed that the β-catenin level was significantly increased in the nuclear fraction of Wnt3a treated cells. At the same time, p27 protein level was significantly increased suggesting a possible interaction between β-catenin and p27 in the cell nucleus (Figures 4C,D).

**FIGURE 5:**
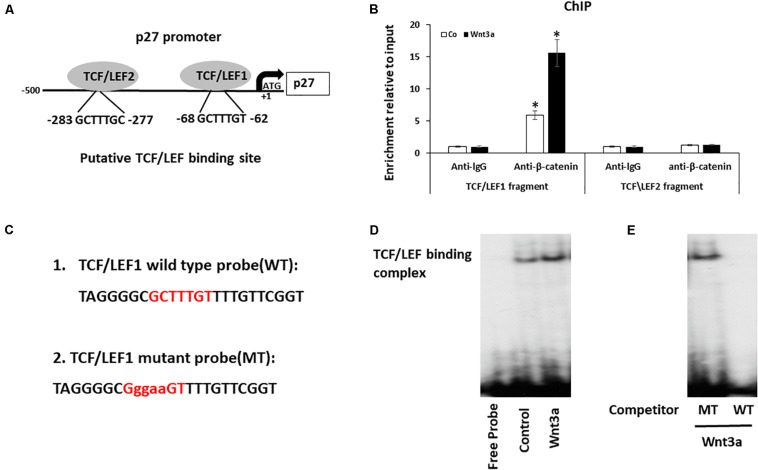
Wnt signaling induces cell cycle inhibitor p27 by direct binding to promoter. **(A)** Analysis of the p27 promoter region and two putative TCF/LEF binding sites were identified. **(B)** Cross-linked chromatin was isolated from cells treated with Wnt3a for 3-days and immunoprecipitated with anti-β-catenin antibody. Enrichment of immunoprecipitated chromatin was quantified by Quantitative PCR (qPCR) of two regions of the p27 promoter, amplicons 1 and 2, the positions of which are indicated in the p27 gene schematic. Results are mean ± SD. All experiments were performed in triplicate. ^∗^*P* < 0.05 vs. anti-lgG controls. **(C)** The ^32^P-ATP radiolabeled oligonucleotides corresponding to the TCF/LEF1 site in p27 promoter were used in Gel-shift assay. **(D)** Gel-shift assay using nuclear extracts isolated from Wnt3a treated cells were incubated with ^32^P−ATP radiolabeled double stranded oligonucleotide spanning the TCF/LEF1 response element. **(E)** Gel-shift assay showed the protein/DNA interaction is competed by self-cold oligo at a 100-fold excess (WT) but not with the cold TCF/LEF1 mutated oligo at a 100-fold excess (MT).

It has been reported that When β-catenin is translocated from the cytoplasm to the nucleus, it forms a complex with the ternary complex factor (TCF) and/or lymphoid enhancer-binding factor (LEF) and stimulates target gene transcription (Dong et al., 2006), we therefore identified the putative TCF/LEF sites in p27 promoter that are responsive for this regulation. Since previous study reported that in human cell lines, the major transcription initiation site is 472 nucleotides upstream of the ATG (Coleman et al., 2001), we focused specifically on this region and identified two 100% matching TCF/LEF consensus binding elements in this fragment of human P27 promoter. One is located between nucleotides −62 and −68, and another one is located between nucleotides −277 and −283 (Figure 5A).

To investigate whether Wnt signaling modulates the expression of p27 by binding to TCF/LEF elements of the p27 promoter, we performed ChIP assays to determine whether a protein-DNA interaction is present at both TCF/LEF binding sites on p27 gene using the nuclear extracts collected from Wnt3a treated BMSCs. Since β-catenin complex is the major chromatin remodeling complex which responds to Wnt3a treatment, antibody against β-catenin was used to pulldown chromatin complex. Our data showed a recruitment of β-catenin at the p27 promoter was observed only in fragment contains TCF/LEF binding site1, but not in fragment targeting TCF/LEF binding site 2 when compared to control lgG. More importantly, Wnt3a treatment further increased β-catenin occupancy on TCF/LEF1 binding site, while no changes were noticed on TCF/LEF2 binding site (Figure 5B).

To confirm the direct binding of β-catenin to the p27 promoter, we performed EMSA using specific probe to target TCF/LEF1 site since we only observed protein-DNA complex formation in this region (Figure 5C). The EMSA results clearly showed that a protein-DNA complex was formed when the nuclear fractions of cells were incubated with the ^32^P-ATP radiolabeled double stranded oligonucleotide spanning the TCF/LEF response element located between -62 and -68 on the p27 promoter. Furthermore, the protein/DNA interaction on the TCF/LEF consensus sequence in the p27 promoter was significantly enhanced when cells were treated with Wnt3a (Figure 5D), and more importantly, this protein/DNA binding is completely abolished after competition with wild-type oligonucleotide, while it remained unchanged after addition of 100 times excess of the TCF/LEF-mutated oligonucleotide (Figure 5E). These results establish the specificity of the Wnt signaling induced DNA binding complex on the p27 promoter, and the requirement of this TCF/LEF binding site1 for this interaction.

## Discussion

Cell culture systems have been widely used in the development of new drugs and drug testing (Langhans, 2018). A majority of drugs used today are primarily screened in cell culture system prior to *in vivo* testing. Therefore, a mature disease-specific cell culture system is useful to identify the underlying mechanisms and evaluate efficacy and predict the toxic potential of drugs. Removal of senescent cells delays aging-related pathologies in mice highlights the therapeutic potential of anti-senescence agonists in treatment of aging-related disease (Baker et al., 2011). Thus, it becomes crucial to develop a cell culture-based model to recapitulate the development of cell aging that could be used to screen anti-senescence and aging compounds.

This study characterized cellular and molecular changes of senescent and apoptotic BMSCs in cell sheet cultures. Our staining of SA-β-Gal, a known marker of cellular aging in different tissue, showed a significant increase of cellular senescence from Day 1 to 4 and Day 7 suggesting a progressive cell aging in high-density cell sheet cultures. Previous reports have shown spontaneous apoptosis can be triggered by an increase of cell density and induced by unknown factors in an autocrine manner (Fiore and Degrassi, 1999). Here, we further monitored apoptosis cells in cell sheet cultures. Our data showed the proportion of cells undergoing apoptosis significantly increased in later stage cultures from Day 4 to 7, but not in early stage cultures from Day 1 to 4. Interestingly, this high cell density culture-induced increase of apoptosis was clearly delayed compared to the quick increase of senescent cells from Day 1 to 4, suggesting more cells enter cellular senescence than apoptosis in an early stage of cell sheet cultures, while more cells enter apoptosis than senescence in later stage cultures.

The cell proliferation assay by BrdU labeling showed a quick stop of cell proliferation at Day 1 cell sheet culture, which clearly confirms that the process of cell contact inhibition was involved in cell sheet culture. This contact inhibition ensures that there is a similar number of cells in sheet cultures from Day 1 to 7. As cell density-dependent apoptosis is often mediated by unknown soluble factors in a conditioned medium (Lund et al., 1996), we next performed ELISA for several inflammatory cytokines that are reported to be responsible for stimulating apoptosis. As expected, we found a significant change in BMSC sheet cultures with a more sustained inflammatory profile in advanced Day 7 MSCs when compared to Day 4 sheet cultures and younger cells at Day 1 evidenced by greater expression of IFNγ, IL-1β, and TNF-α levels. Moreover, our PCR data clearly showed that Day 7 MSC sheet cultures present a trend for a decreased expression of Bcl-2, Bax and for an increased production of caspase 3 mRNA levels. These data strongly suggest cell sheet culture-induced apoptosis is regulated by increased inflammatory cytokines. Given that senescent cells actively secrete a group of cytokines, chemokines, and matrix-remodeling enzymes known as the SASP (Coppé et al., 2010), we speculated senescent cells might be the major source of pro-inflammatory cytokines and chemokines, that drive a portion of healthy cells to enter apoptosis in cell sheet culture. The relevance of SASP and apoptosis was further supported by gene expression data showing significant changes in the secretory profile of Day 7 aging MSCs in cell sheet cultures.

Because p53/p21 and p16 proteins have been identified as the main regulators of the senescence program (Ben-Porath and Weinberg, 2005), we further measured their expression to characterize the senescent state in cell sheet cultures. Our results confirmed the increase of p53/p21 and p16 pathway in aging cell sheet cultures. Interestingly, we also found a quick increase of activation in the p16 at early stage of cultures from Day 1 to 4. In contrast, a more dramatic increase of p53/p21 was observed in later stage of cultures from Day 4 to 7. Because the activation pattern of p16 and p53/p21 is similar to the increased pattern of cellular senescence and apoptosis, respectively, we speculated p16 is a related to cell senescence, while p53/p21 is related to cell apoptosis. Moreover, a rapid decrease in the expression of cyclin D1 followed by lower expression of p53/p21 and p16 was also observed in early stage of cell sheet cultures further confirming the cell aging *in vitro*. Additionally, other imbalanced factors, such as the accumulation of ROS, are also involved in the induction and/or the development of senescence and apoptosis (Colavitti and Finkel, 2005; Sies, 2015). Consistent with the earlier report, our work found ROS level quickly increased from Day 1 cells in low cellular stress to Day 4 cells in mild cellular stress and Day 7 cells in high cellular stress. At the same time, we noticed that more apoptotic cells were observed in more aging BMSCs at Day 7 compared to younger BMSCs at Day 1 and Day 4. This suggests at a low ROS level, the stromal cells in sheet cultures are more susceptible to senescence than apoptosis, while at a high ROS level, the cells are more susceptible to apoptosis than senescence.

p27 is a member of the Cip/Kip family of CDK inhibitory proteins, which plays a pivotal role in the control of cell proliferation, differentiation, and apoptosis. p27 is a negative regulator of the protein kinase CDK2/cyclin E and can induce cell quiescent by blocking transition from G1 phase to S phase of the cell cycle via inhibition of cyclin-CDK complexes. In contrast to other cell cycle inhibitors with increased expression in stromal cell sheet culture, we observed a significant decrease of p27 expression in stromal cell sheet culture. More importantly, Wnt signaling effector β-catenin showed a similar expression pattern to p27 suggesting a possible interaction between these two factors. A previous study also supports that Wnt signaling might be a predominant factor that could be used to overcome extensive passaging-induced cell aging in long-term cultured MSCs by directly intervening in the proliferative capacity and MSC senescence (Jeoung et al., 2015). In this study, we further discovered the underlying mechanism by using a unique cell sheet culture-induced cell aging system. As we expected, 3-day Wnt3a treatment significantly reduced senescence in stromal cell sheet culture with a rapid increase of p27 expression suggesting more cells entered quiescence instead of senescence. Promoter analysis further identified two putative TCF/LEF binding sites in p27 promoter region. Surprisingly, no protein-DNA complex formation was noticed when the oligonucleotide spanning nucleotides -277 and -283 were used, indicating that the active effect of catenin on the p27 promoter is conferred by the TCF/LEF binding element located between -62 and -68, not the one located between -277 and -283. Initiation of stromal cell aging in our cell sheet culture involves cell cycle arrest with increased expression of cell cycle inhibitors p16, p21 and p53, while decreased expression was noticed on cell cycle inhibitor p27 suggesting a different role of p27 to other cell cycle inhibitors. Our Wnt3a treatment study further indicates p27 induced cell quiescent may also plays important role to reduce cell senescence in cultures. Since p27 is a major target of Wnt signaling, we speculate that Wnt3a treatment induces cell cycle arrest in stromal cell sheet cultures, which leads more cells entering quiescent stage instead of entering cell senescence and apoptosis pathway.

**FIGURE 6:**
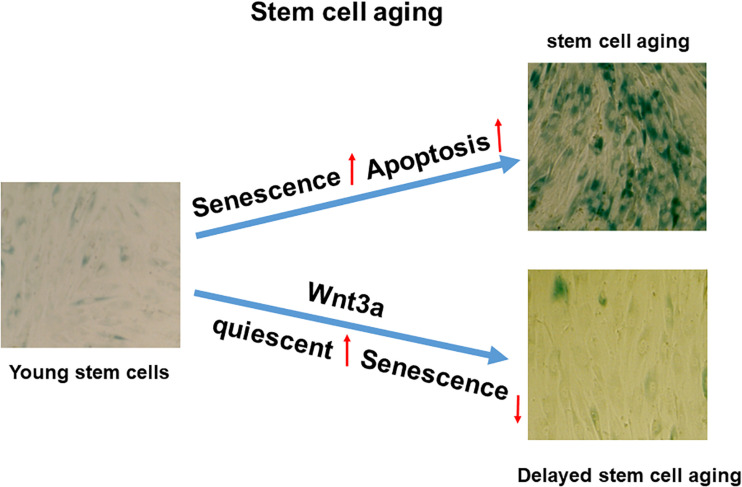
Proposed working model in cell sheet culture-induced stromal cell aging. High-density cell sheet culture induces stromal cell aging via senescence and apoptosis pathways, while Wnt signaling activation by Wnt3a prevents cell entering senescence by switching to quiescent cells.

In this study, we showed the BMSCs in cell sheet cultures spontaneously undergo senescence and subsequent apoptosis without external stimulation. Although cell sheet technology has been used widely for tissue regeneration and repair, particularly MSC sheet tissue engineering becomes an important tool for regenerative medicine, while to avoid cell death in culture, most of these cell sheets were generated in monolayer within a very short of period. Therefore, it is necessary to develop a novel cell sheet culture that could be used to generate multiple layer cell sheets without causing any cell death. In this study, we demonstrated cell sheet culture-induced spontaneous senescence and apoptosis could be reduced by activation of Wnt signaling in a p27 dependent manner. Finally, our work validated that high-density stromal cell sheet culture is an *in vitro* stromal cell aging model with quickly developed senescence and apoptosis, while Wnt signaling activation can be used to switch cells from entering senescence to quiescent stage (Figure 6). While our finding in this study is significant, some limitations are still identified. First, we only studied the cell sheet culture induced senescence and apoptosis in BMSCs. Whether that is the case in other cell types or stromal cells derived from other organs was not analyzed. Second, the effect of Wnt3a on the BMSC capacity to differentiate into specific tissue-lineage phenotypes, including chondrogenic-, osteogenic- and adipogenic differentiation was not included in this study. Therefore, more experiments will be performed in a separated study in the future.

## Data Availability Statement

The raw data supporting the conclusions of this article will be made available by the authors, without undue reservation, to any qualified researcher.

## Author Contributions

YT designed all the experiments. YT and YX supervised the research. YX performed the most experiments and data collection, along with YT, DT, HZ, ZL, and XS generated the immunofluorescence data. YX, YT, and YD obtained the funding. YX wrote the manuscript. YT and YD analyzed data and edited the manuscript. All authors contributed to the article and approved the submitted version.

## Conflict of Interest

The authors declare that the research was conducted in the absence of any commercial or financial relationships that could be construed as a potential conflict of interest.
